# AlkaPhos: a novel fluorescent probe as a potential point-of-care diagnostic tool to estimate recurrence risk of meningiomas

**DOI:** 10.1007/s10143-024-03172-8

**Published:** 2025-01-08

**Authors:** Sina Hemmer, Xin Hui, Julia Draeger, Johannes Menges, Eva C. Schwarz, Arne Wrede, Joachim Oertel, Lars Kaestner, Gregor Jung, Steffi Urbschat

**Affiliations:** 1 Department of Neurosurgery, Saarland University Medical Center, Homburg, Germany; 2 Biophysical Chemistry, Saarland University, Saarbrücken, Germany; 3 Theoretical Medicine and Biosciences, Saarland University, Homburg, Germany; 4 Biophysics, Center for Integrative Physiology and Molecular Medicine, Saarland University, Homburg, Germany; 5 Institute for Neuropathology, Saarland University Medical Center, Homburg, Germany; 6 Experimental Physics, Saarland University, Saarbrücken, Germany

**Keywords:** Meningioma recurrence, Deletion of chromosome 1p, Alkaline phosphatase detection, Primary meningioma cell cultures, Fluorescence microscopy

## Abstract

Deletion of the short arm of chromosome 1 (1p) increases recurrence rates in meningiomas by up to 33%, regardless of tumor grade, correlating with absence of intracellular alkaline phosphatase enzyme activity. Current screening methods for 1p deletion like fluorescence in situ hybridization (FISH) and loss of heterozygosity (LOH) analysis are resource-intensive. This study evaluated AlkaPhos, a novel fluorescent probe, for detecting alkaline phosphatase in meningioma cells and compared findings with FISH, LOH, and histochemical analysis. AlkaPhos sensitivity in detecting alkaline phosphatase on BEN-MEN-1 cells and primary meningioma cultures was assessed via microscopic fluorescent ratio measurements. FISH and LOH were conducted on the same tumors to detect 1p deletions. Histochemical analysis served as a reference. AlkaPhos results were compared with FISH, LOH, and histochemical analysis. AlkaPhos effectively indicated alkaline phosphatase activity in BEN-MEN-1 cells and correctly identified 1p deletion in 8/14 primary meningioma cultures, matching FISH and LOH findings, respectively. AlkaPhos showed potential superiority over histochemical analysis in identifying tumors with 1p deletion and LOH of 1p. AlkaPhos bears potential as a future diagnostic tool for identifying alkaline phosphatase absence in meningiomas, indicative of 1p deletion. Further evaluation on a larger sample size is necessary for routine clinical application.

**Supplementary Information:**

The online version contains supplementary material available at 10.1007/s10143-024-03172-8.

## Introduction

Meningiomas are mostly benign tumors of the central nervous system that can be cured in most cases if complete surgical resection is achieved [9, 22, 32]. Histologically, the current World Health Organization (WHO) classification distinguishes between grades 1, 2 and 3 as benign, atypical and anaplastic meningiomas, respectively [22]. Meningiomas of higher WHO grades exhibit an increased risk of recurrence despite surgical resection. While an overall recurrence rate of about 6% after surgery has been reported for all meningiomas, the risk is as high as 30% in anaplastic meningiomas [15, 18, 21]. Although completely resected and primarily classified benign, a subset of WHO grade 1 meningiomas still recur [5, 15, 18]. In this context, specific chromosomal aberrations and desoxyribonucleic acid (DNA) methylations have been investigated. Aberrations of chromosome 1p, 14, 17q and 18 have been demonstrated to correlate with tumor recurrence and progression of meningiomas [12, 18]. Notably, the only chromosomal marker for meningioma recurrence independent from the WHO grading system is the deletion of the short arm of chromosome 1 (1p-) [17, 19, 21]. Recurrence rates among meningiomas with deletion of 1p have reported to be up to 33%, compared to 8% for meningiomas without this aberration [18]. Hence, screening for 1p deletion in meningiomas is a crucial diagnostic element to identify patients at risk for meningioma recurrence. Molecular cytogenetic testing for 1p deletion is preferably performed using fluorescence in situ hybridization (FISH) or loss of heterozygosity (LOH) [20, 23]. 

On the chromosomal segment 1p36.1-p34, the *alkaline phosphatase* gene is located as a single-copy gene. Cytogenetic confirmation of 1p deletion via FISH highly correlates with the absence of histochemical alkaline phosphatase activity, and loss of alkaline phosphatase activity has been identified as a histochemical marker for poor meningioma differentiation [23, 24]. 

However, FISH, LOH and histochemical analysis are infrastructure-demanding and resource-consuming. In contrast, fluorescence microscopy represents a convenient alternative since a timely, reliable and sensitive analysis can be performed using an appropriate fluorescent probe.

A fluorescent probe based on the hydroxylated pyrene scaffold for assessing phosphatase activity was recently introduced [8]. Hydroxypyrene derivatives are characterized by their bright fluorescence in the visible range and can be easily synthesized [7]. A previously established phosphorylation method was transferred to one of these derivatives yielding AlkaPhos (Bis(methylene)diacetate-(3,6,8-tris(*N*-methoxy-N-methylsulfamoyl)pyren-1-yl)phosphate [8]. Due to its dual emission characteristic, this new fluorescent probe enables the distinction between substrate and product.

Studying primary meningioma cell cultures, this study sought to evaluate if AlkaPhos is a suitable tool to detect loss of alkaline phosphatase activity in meningiomas and correlate observations with conventional molecular genetic testing (FISH and LOH) and histochemical analysis.

## Materials and methods

### Study design

The study was approved by the Ethics Committee of the Saarland Medical Association (Number 178/07). Written informed consent was obtained from all patients.

**Fig. 1:**
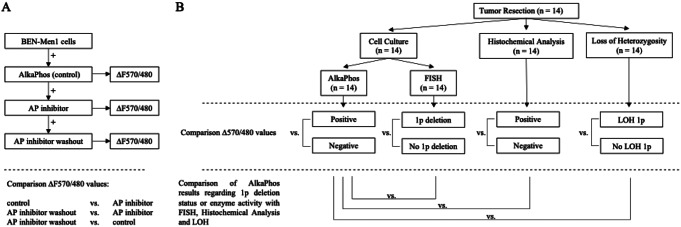
Flow chart overview of the two-phase study design. (**A**) Evaluation of AlkaPhos on BEN-MEN-1 cell line: The fluorescence ratio ∆F570/480 was measured on cells treated with AlkaPhos as a control, the same cells treated with an alkaline phosphatase (AP) inhibitor, and the same cells again after washout of the AP inhibitor. Comparison of ∆F570/480 values were conducted between the control, AP inhibitor treatment, and AP inhibitor washout groups. Study design for analysis on BEN-Men1 cell cultures. (**B**) Assessment of AlkaPhos in Primary Meningioma Cell Cultures: Tumor samples (*n* = 14) were collected during surgical resection, followed by cell culture establishment and analysis via AlkaPhos, fluorescence in situ hybridization (FISH), histochemical detection of alkaline phosphatase activity, and loss of heterozygosity (LOH) testing. The ΔF570/480 values were compared between the result groups of each testing modality (AlkaPhos positive vs. negative; FISH 1p deletion vs. no 1p deletion; histochemical analysis positive vs. negative enzyme activity; LOH analysis LOH 1p vs. no LOH 1p). Analytical comparisons between AlkaPhos results and conventional testing methods (FISH, histochemical analysis, and LOH) were conducted to evaluate the diagnostic potential of AlkaPhos for detecting 1p deletion and alkaline phosphatase activity in meningioma cells

The study was structured in two phases. In the first phase, the specificity of AlkaPhos in determining alkaline phosphatase activity was evaluated on the human meningioma cell line BEN-MEN-1 without chromosomal aberrations of chromosome 1p. (Fig. 1A) In chronological order, AlkaPhos and an alkaline phosphatase inhibitor (AP inhibitor) were added to the cell cultures, before a washout of the AP inhibitor was performed. After each step, fluorescence ratios ΔF570/480 were measured and results were compared.

In the second phase of the study, AlkaPhos was validated on primary meningioma cell cultures. (Fig. 1B) Meningioma tumor tissue was obtained during standard-of-care tumor resection and tumor samples from each case were utilized for cell culture, histochemical analysis and LOH testing. The primary cell cultures were subsequently analyzed using FISH and AlkaPhos.

After application of AlkaPhos to the cell cultures, fluorescence ratios (ΔF570/480) were measured for each sample. The ΔF570/480 values were compared across groups based on the outcomes of FISH, histochemical analysis and LOH (e.g., ΔF570/480 values from samples with 1p deletion detected by FISH were compared to those without 1p deletion).

In the final step, the performance of AlkaPhos in detecting 1p deletions was compared with the results obtained from FISH, histochemical analysis and LOH testing.

### Preparation of cell cultures

Primary meningioma cell cultures were prepared with freshly resected minced tumor fragments of 14 histologically confirmed meningiomas (8x WHO grade 1, 5x WHO grade 2, 1x WHO grade 3) suspended in Dulbecco’s Modified Eagle Medium (DMEM) and distributed into separate cell culture flasks (tumors T2 and T3 represent two separately located, simultaneously resected tumors from one patient).

Human meningioma cell line BEN-MEN-1 [26] cells (Number ACC 599, German Collection of Microorganisms and Cell Cultures, DSMZ, Braunschweig, Germany) as well as primary tumor cell cultures were cultured in a complete cell culture medium (Dulbecco’s Modified Eagle Medium with 10% fetal bovine serum and 1% Non- Essential Amino Acids, ThermoFisher, Germany), incubated at 37 °C in a humidified atmosphere containing 5% CO_2_ and processed as described before [20]. A total of 50,000 cells were seeded on a coverslip in each well of 12-well plates 24 h prior to further analysis. In preparation of the fluorescence in situ hybridization, parts of the primary tumor cell cultures were fixed in a cell suspension and dropped onto object slides as described by *Lerner et al.* [20]

### Fluorescence in situ hybridization (FISH)

FISH for the chromosomal regions 1p and 22q after a modified protocol of Pinkel et *al.* was performed on primary meningioma cell cultures using DNA probes from MetaSystems [25]. Samples were analyzed using a fluorescence microscope. At least 200 nuclei of each tumor were analyzed according to the criteria of Hopman et *al.* [16] The cut-off value for alterations was set at 10%.

### Loss of heterozygosity (LOH)

#### Microsatellite PCR

Loss of heterozygosity analysis was performed using primers flanking microsatellites as routinely done in the department of neuropathology. For the PCR reaction 1.3 µl DNA, 1.0 µl primer mix (both forward and revers primer at a concentration of 20 µM, (Eurofins Genomic, Ebersberg, Germany), 10.2 µl water, and 12.5 µl HotStarTaq Master Mix Kit (Qiagen, Hilden, Germany) were mixed (25.0 µl total reaction volume). The PCR reaction was performed according to the protocol developed by Hartmann et *al.* [10] The microsatellites used to detect losses in 1p are distributed across the affected chromosomal arms and their location is listed in Table 1.

**Table 1:** Locations of the microsatellites (STS marker ID, if previously reported) used to detect losses in 1p, distributed across the affected chromosomal arm (chromosomal location)

STS marker ID, if previously reported	Chromosomal location
1 D1S 1608	1p36.32
2 -	1p36.11
3 D1S 1161	1p35.2
4 D1S 1184	1p31.1

#### LOH analysis

PCR products were visualized on high-resolution Spreadex^®^ EL 800 Wide Mini gels (AL-Diagnostic GmbH, Amstetten, Austria) using an Origins electrophoresis system (AL-Diagnostic GmbH, Amstetten, Austria) and SYBR^TM^-Gold Nucleic Acid Gel Stain (ThermoFisher Scientific, Dreieich, Germany). We conducted gel electrophoresis for 90 min at 120 V in TRIS-acetate-EDTA-buffer (#42548.01, TAE buffer, SERVA, Heidelberg, Germany) at 54 °C. Afterwards, gels were washed once with distilled water, stained with staining solution for 30 min [final buffer concentration 0.8x TAE (#42548.01, SERVA, Heidelberg, Germany), 0.8x Destaining Solution (#3037.01, SERVA, Heidelberg, Germany), 2x SYBR™ Gold] and washed again with distilled water. We documented results using the ‘EOS Utility’ program (Canon, Tokio, Japan).

### Histochemical analysis of alkaline phosphatase

Frozen tumor sections of 7–8 μm thickness were mounted on glass slides, dried for 20 min and then exposed to alkaline phosphatase solution using Naphthol-AS-MX-phosphate as a chromogen. After counterstaining with hematoxylin, slides were dried and afterwards mounted with permanent mounting medium (Entelan, Merck KGaA, Darmstadt, Germany). The enzyme reaction for alkaline phosphatase was considered positive when any cytoplasmic staining was detectable. Endothelial cells of blood vessels served as an internal positive control. The tumors were classified into three different groups concerning the staining pattern of the tumor according to the criteria of Bouvier et *al* [4]. Group 1: uniform expression of alkaline phosphatase by all tumor cells; group 2: heterogeneous pattern of alkaline phosphatase expression with loss of enzymatic activity in large areas of the tumor tissue (> 50%) but preserved activity in other areas; group 3: complete lack of alkaline phosphatase expression in tumor cells. Tumors of groups 1 and 2 were rated as “positive” for the presence of alkaline phosphatase.

### AlkaPhos characteristics

AlkaPhos (Bis(methylene)-diacetate-(3,6,8-tris(*N*-methoxy-*N*-methylsulfamoyl)-pyren-1-yl)phosphate) was synthesized similarly as described in Finkler et *al* [7, 8]. Structural formulas of AlkaPhos **(1)**, its salt form **(2)** and the product **(3)**, as well as information on synthesis, intracellular metabolization and determination of fluorescence spectra of AlkaPhos are provided in the Supplemental material.

**Fig. 2:**
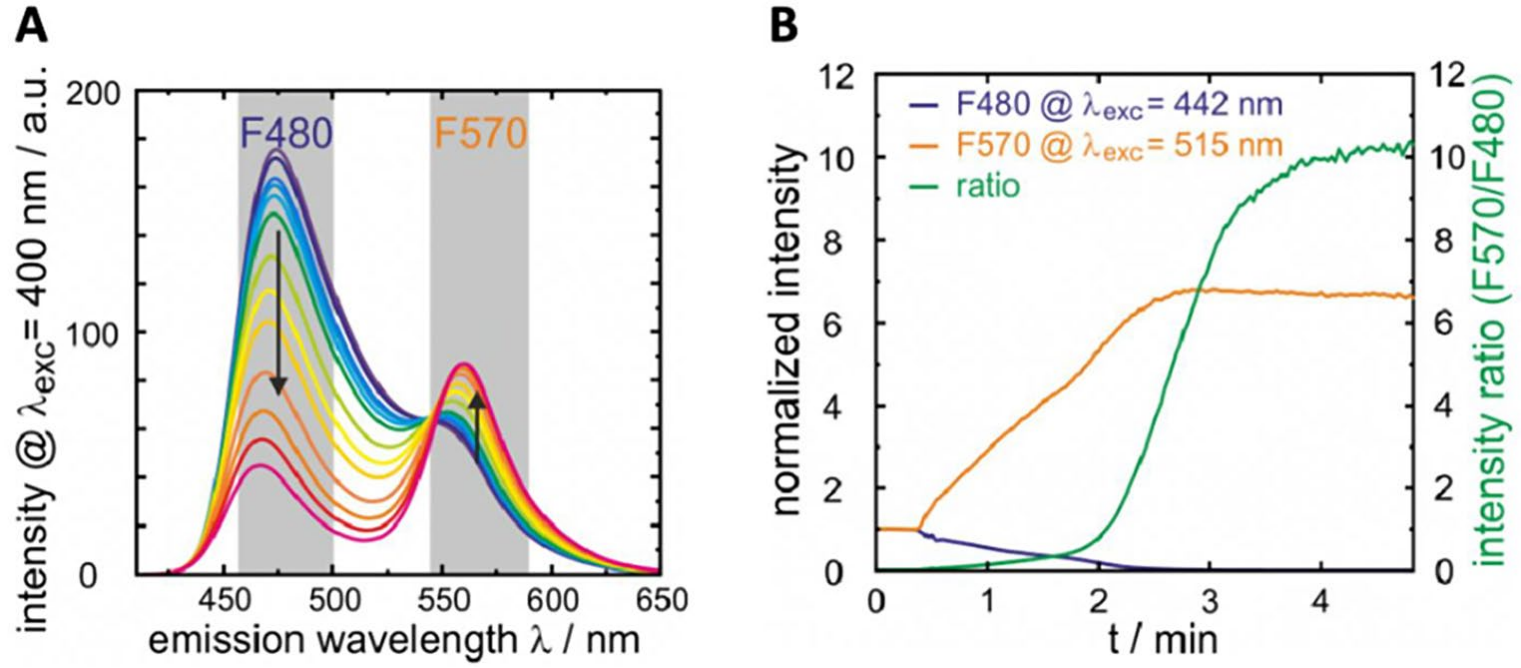
(**A**) Fluorescence spectra of AlkaPhos with increasing dephosphorylation indicated by the black arrows. (**B**) Time course of dephosphorylation of AlkaPhos in solution measured with the same microscope settings as used for Figs. 5 and 6. The two spectral channels (F480 and F570) corresponding to emission filter settings as indicated by the grey areas in (**A**) and laser excitation wavelengths (ƛ_exc_) of 442 nm and 515 nm, respectively

AlkaPhos (**1)** is intracellularly transformed into the actual phosphatase substrate (**2)**. The substrate (**2)** is converted into (**3)** in the presence of alkaline phosphatase. Both the substrates (**1)** or (**2)**, respectively, and the product (**3)** can be easily distinguished by their electronic spectra ((**1**): λ_abs_ = 407 nm; λ_em_ = 473 nm; (**2**): λ_abs_ = 416 nm; λ_em_ = 477 nm; (**3**): λ_abs_ = 510 nm; λ_em_ = 557 nm at pH > 6) [7]. Two excitation (λ_exc_ = 442 nm and 515 nm) images are recorded at the two spectrally separated channels F480and F570 as depicted in Fig. 2 using confocal microscopy as previously described and outlined in the *supplemental material* [1]. The fluorescence image ratio ΔF570/480 was calculated by ImageJ (National Institute of Health, USA, Version 2.14.0) after background correction and served as verification of alkaline phosphatase presence.

### Specificity of AlkaPhos in meningioma cell line BEN-MEN-1

In order to verify the ability of AlkaPhos to specifically detect alkaline phosphatase activity in vitro, AlkaPhos was tested on the meningioma cell line BEN-MEN-1 with an intact chromosome 1p as a positive control. The alkaline phosphatase inhibitor L-p-bromotetramisole oxalate was used as an internal negative control.

The fluorescence ratio ΔF570/480 was measured as described above before and after incubation of the BEN-MEN-1 cells in Tyrode’s solution (135 mM NaCl, 5.4 mM KCl, 2 mM MgCl_2_, 1.8 mM CaCl_2_, 10 mM glucose, 10 mM Hepes, pH at 7.35) with 4 μM AlkaPhos for 5 min at room temperature. Measurements were repeated after additional co-incubation with 200 µM of L-p-bromotetramisole oxalate. Afterwards, a wash-out of L-p-bromotetramisole oxalate was performed and the fluorescence ratio was measured again. The difference of the mean fluorescence ratios before and after applying L-p-bromotetramisole and also after wash-out of L-p-bromotetramisole were compared to evaluate the specificity of AlkaPhos for alkaline phosphatase detection.

### AlkaPhos on primary meningioma cell cultures

In order to assess the ability of AlkaPhos to detect alkaline phosphatase activity in meningiomas, cells from primary meningioma cell cultures were seeded on coverslips and bathed in 500 µL Tyrode’s solution before they were mounted on the confocal microscope system. The fluorescence ratio ΔF570/480 was measured as described above.

AlkaPhos was prepared in Tyrode’s solution at 8 µM as working solution. After adding 500 µL AlkaPhos working solution and an incubation time of 5 min at room temperature on each coverslip, repeat measurement of the fluorescence ratio ΔF570/480 was executed. For every tumor cell culture, fluorescence ratios of 60 to 80 cells were record. Mean fluorescence values for every tumor were calculated. The threshold for a positive AlkaPhos reaction was calculated based on the mean ΔF570/480 values of all cell cultures. Cell cultures with a mean fluorescence ratio ΔF570/480 > 0.426 ware categorized as a positive reaction.

### Statistics

Statistical analysis was performed in the Prism 9.0.0 software (GraphPad, USA). Differences between two groups were analyzed by either the Mann-Whitney-U test or unpaired t-test after testing for Gaussian distribution with the Shapiro-Wilk test. Statistical significance was accepted for p-values < 0.05. To compare three non-normally distributed groups, Kruskal Wallis test was performed. Post-hoc testing was performed with Mann-Whitney-U test and Bonferroni correction.

## Results

### Fluorescence in situ hybridization

**Fig. 3:**
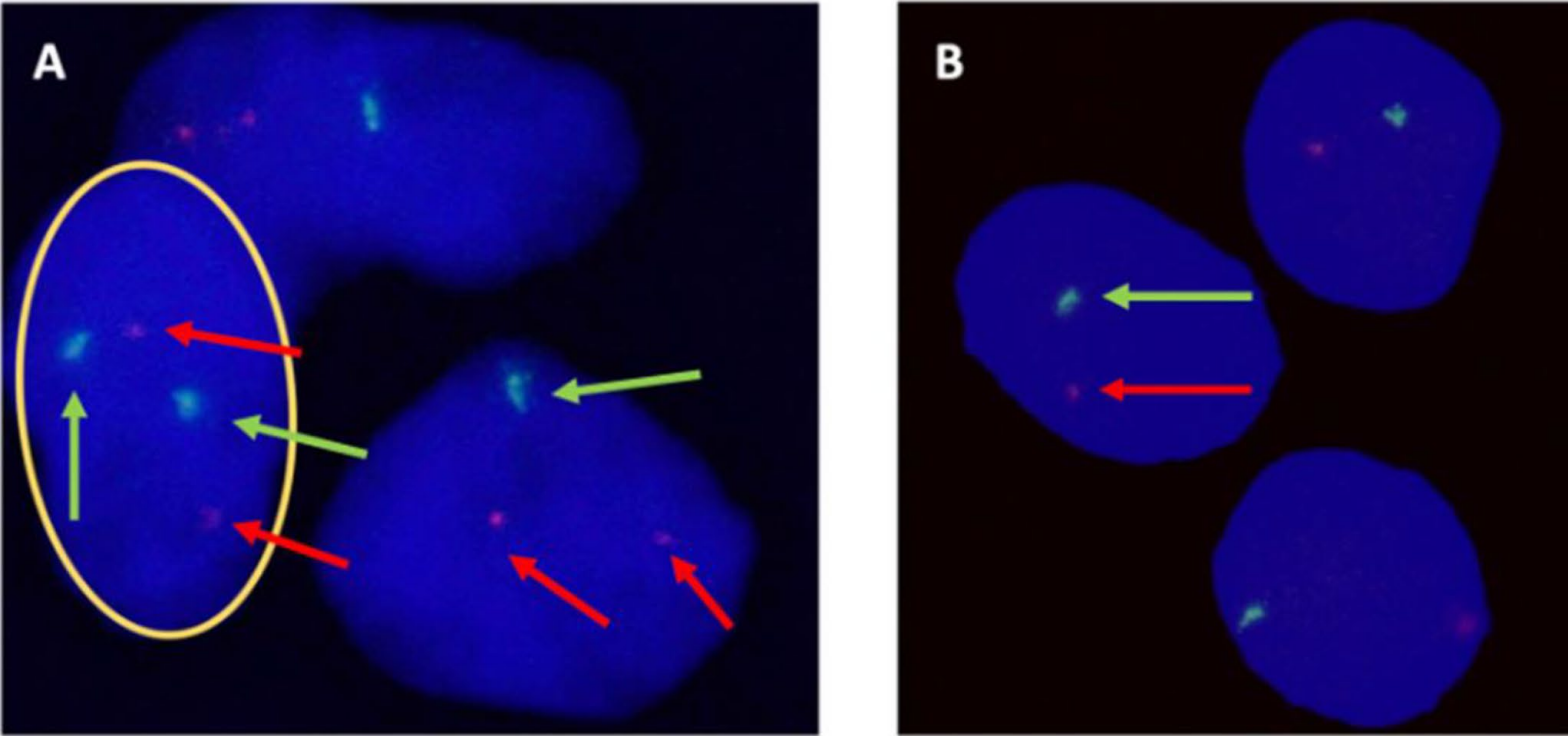
Representative micrographs of primary meningioma cell cultures using fluorescence in situ hybridization with probes 1p36 (red) and 22q11 (green). (**A**) Highlighted cell with intact 1p and 22q (two red and two green signals). Two cells with intact 1p and loss of 22q (two red signals and one green signal). Scale bar equals 2 μm (**B**) Cells with deletion of 1p and loss of 22q (one green and one red signal). Scale bar equals 5 μm

Primary cell cultures of 14 different meningiomas were examined with FISH. Deletion of 22q was detected in 9/14 of the primary cell cultures. Deletion of 1p was present in 8/14 of the cell cultures (Table 2). Figure 3 portrays representative micrographs of FISH diagnostics.

**Table 2:** Results of the AlkaPhos-testing, fluorescence in situ hybridization (FISH), loss of heterozygosity (LOH) and histological analysis (ALP histo) for tumors T1-14. WHO grades 1–3, FISH 22q: 1 = 22q deletion. 0 = no 22q deletion. FISH 1p: 1 = 1p deletion. 0 = no 1p deletion. Neg = alkaline phosphatase absent. Pos = alkaline phosphatase present. Mean fluorescence: Δ 570/480 values

Tumor	WHO grade	FISH 22q	FISH 1p	LOH	AlkaPhos	ALP Histo	Mean Fluorescence
T1	1	0	0	0	pos	pos	0.865
T2	2	1	1	1	neg	pos	0.355
T3	2	1	1	1	neg	pos	0.39
T4	3	1	1	1	neg	neg	0.292
T5	1	0	0	0	pos	pos	0.746
T6	1	1	1	0	neg	neg	0.339
T7	1	1	1	1	neg	neg	0.253
T8	2	1	1	1	neg	neg	0.423
T9	1	1	0	0	pos	pos	0.801
T10	2	1	1	1	neg	neg	0.285
T11	1	0	0	0	neg	pos	0.325
T12	1	1	0	0	neg	neg	0.306
T13	1	0	0	0	neg	pos	0.29
T14	2	0	1	1	neg	neg	0.296

### Loss of heterozygosity (LOH)

Loss of heterozygosity (LOH) analysis was performed on 14 meningioma samples. LOH for chromosome 22q was confirmed in 12/14 of tumors. For chromosome 1p, LOH was detected in 7/14 of tumors (Table 2).

### Histochemical analysis of alkaline phosphatase

**Fig. 4:**
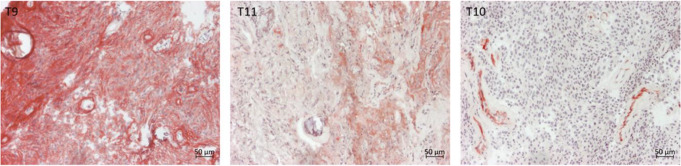
Alkaline phosphatase staining on instantaneous sections. T9: uniform expression of alkaline phosphatase, T11 heterogenous expression of alkaline phosphatase T10: negative alkaline phosphatase reaction. Micrographs were recorded with x100 magnification. The scale bar on the bottom righthand side equals 50 μm

The presence of alkaline phosphatase was detected in 7/14 samples (Table 2). Exemplary microscopic images of a negative, intermediate and positive sample are given in Fig. 4.

### Specificity of AlkaPhos in meningioma cell line

After incubation of BEN-MEN-1 cell cultures with AlkaPhos, a strong fluorescence with a median fluorescence ratio ΔF570/480 of 1.43 was detected (Table 3; Fig. 3A).

**Fig. 5:**
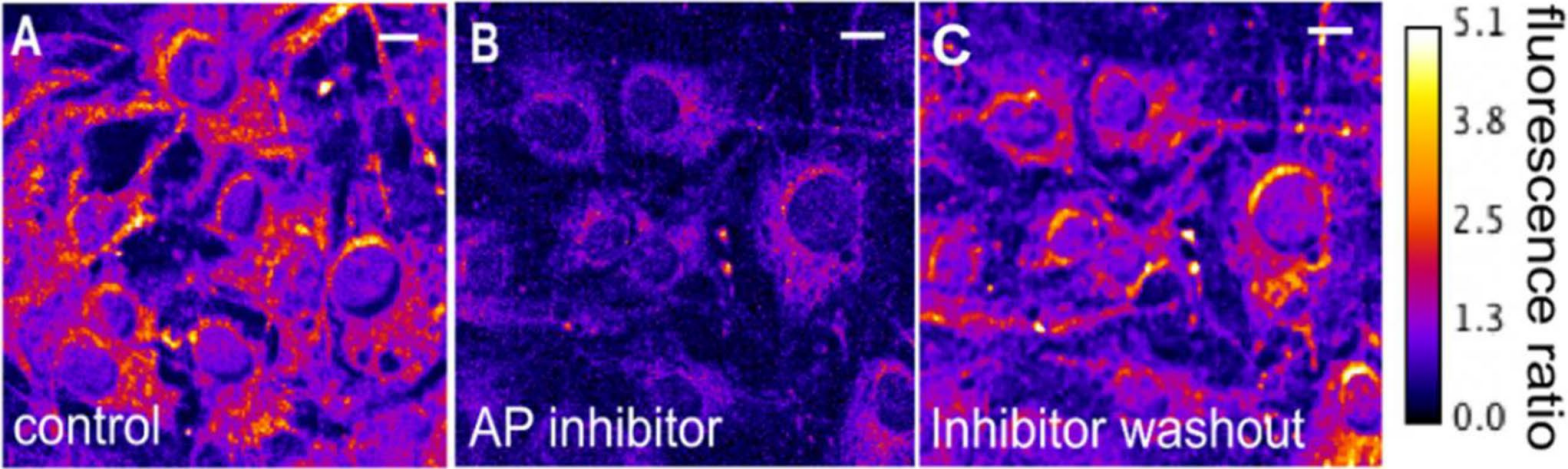
The color wedge(right) represents the color-coding of the fluorescence ratio as indicated. The scale bar is 10 μm in all images. (**A**) The fluorescence ratio (F570/480) image of BEN-MEN-1 cells after 4 µM AlkaPhos incubation in tyrode solution for 5 min room temperature. (**B**) The fluorescence ratio image of BEN-MEN-1 cells after additional co-incubation with 200 µM of the alkaline phosphatase (AP) inhibitor, L-p-bromotetramisole oxalate. (**C**) When washing the alkaline phosphatase inhibitor out, the fluorescence ratio increased again. The scale bar in the upper right corner equals 10 μm in all images

The signal significantly decreased to a median fluorescence ratio of 0.54 ΔF570/480 after the application of the alkaline phosphatase inhibitor L-p-bromotetramisole (Table 3; Fig. 3B). After a wash-out of the alkaline phosphatase inhibitor, the fluorescence ratio increased again to a median fluorescence ratio of 1.539 ΔF570/480 (Table 3; Fig. 5C).

Normal distribution could not be assumed based on the results of Shapiro-Wilk test (control W = 0.82, *p* < 0.0001; AP inhibitor W = 0.94, *p* = 0.0002; AP inhibitor washout W = 0.97, *p* = 0.006) Kruskal Wallis test indicated a difference between the three non-normally distributed groups (H(2) = 164.06, *p* < 0.001). Mann-Whitney-U test with a post-hoc Bonferroni correction indicated a significant difference (each *p* < 0.001) of the median fluorescence ratio ΔF570/480 before and after applying the alkaline phosphatase inhibitor and after the wash-out of the alkaline phosphatase inhibitor, respectively (Table 3). The phosphatase activity could be reversibly inhibited by the alkaline phosphatase inhibitor L-p-bromotetramisole oxalate. The fluorescence signal of AlkaPhos specifically indicates the presence of alkaline phosphatase in BEN-MEN-1 cells.

**Table 3:** Comparison of median fluorescence ratio values ΔF570/480 for BEN-MEN-1 cells after incubation with AlkaPhos (control) (n = 178), after application of the alkaline phosphatase inhibitor L-p-bromotetramisole (AP inhibitor) (n = 113) and after washout of L-p-bromotetramisole (AP inhibitor washout) (n = 144). n = number of measurements; U = value of Mann-Whitney-U

BEN-Men1 cells	Median fluorescence ratio ΔF570/480	n	U	p value
control	1.43	178	1326	<0.001
AP inhibitor	0.54	113		
AP inhibitor washout	1.539	144	1875	<0.001
AP inhibitor	0.54	113		
AP inhibitor washout	1.539	144	12475	0.68
control	1.43	178		

### AlkaPhos on primary meningioma cell cultures

**Fig. 6:**
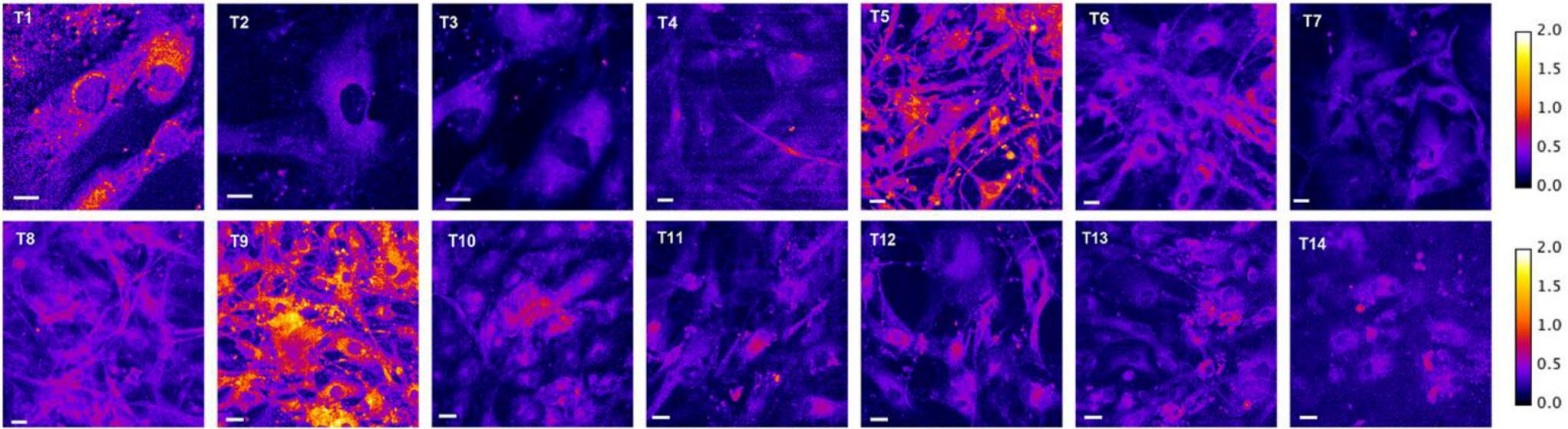
The fluorescence ratio image of cells from tumors T1-14 after 4 µM AlkaPhos incubation for 5 min at room temperature. The color wedges on the righthand side represent the color-coding of the fluorescence ratio as indicated. The scale bar is 10 μm in all images

Fluorescence measurement after application of AlkaPhos was performed on 14 primary meningioma cell cultures. In 11/14 cultures, AlkaPhos indicated the absence of alkaline phosphatase activity, whereas the presence of alkaline phosphatase was indicated in 3/14 samples (Table 2; Fig. 6).

**Fig. 7:**
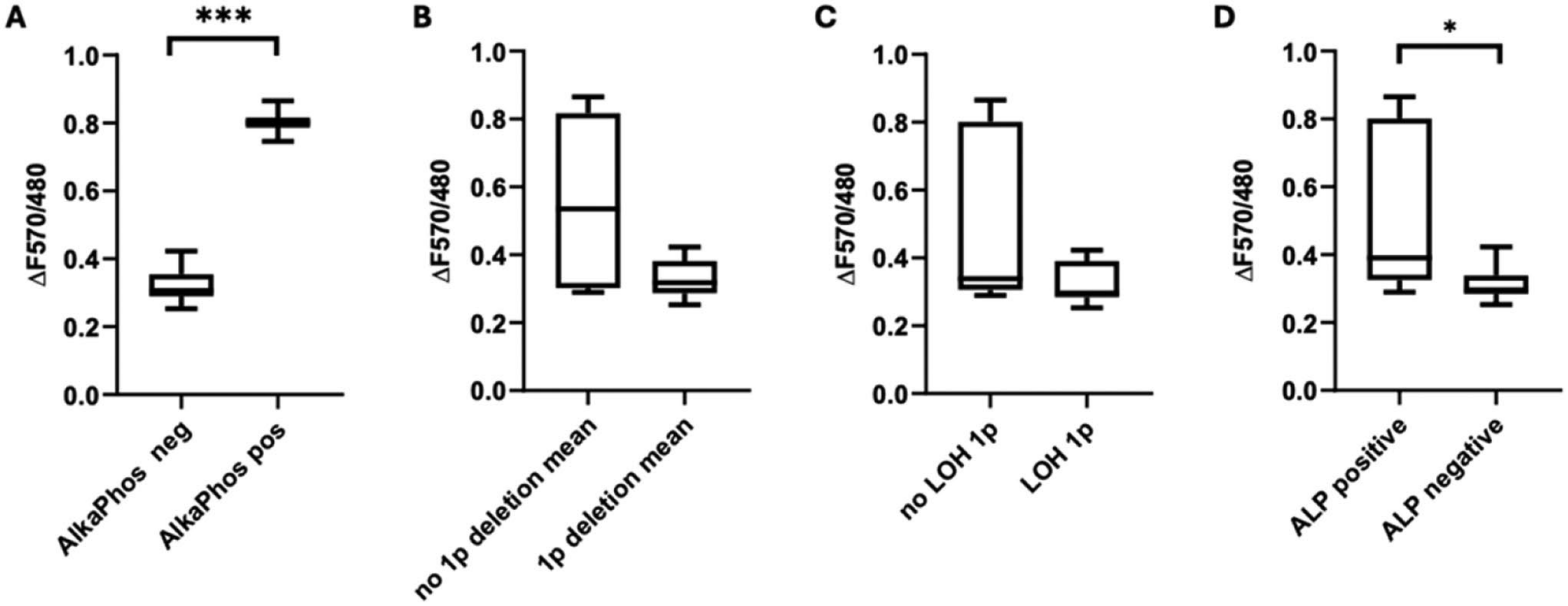
(**A**) Comparison of mean fluorescence values between AlkaPhos negative (*n* = 11) and AlkaPhos positive (*n* = 3) tumors, (p = < 0.0001) asterisks marks statistical significance; (**B**) Comparison of median fluorescence values between tumors with (*n* = 8) and without (*n* = 6) 1p deletion in FISH (*p* = 0.22); (**C**) Comparison of median fluorescence values between tumors with (*n* = 7) and without (*n* = 7) LOH of 1p (*p* = 0.20); (**D**) Comparison of mean fluorescence values between tumors that were tested positive (*n* = 7) and negative (*n* = 7) for alkaline phosphatase activity by conventional histochemical analysis, (*p* = 0.03) asterisk marks statistical significance

Mean fluorescence ratios ΔF570/480 of positive and negative results for presence of alkaline phosphatase were 0.80 (SD ± 0.05) and 0.32 (SD ± 0.05), respectively. Normal distribution was assumed based on the results of Shapiro-Wilk test (negative samples W = 0.93, *p* = 0.46; positive samples W = 0.99 *p* = 0.91). A significant difference in fluorescence ratio between positive and negative samples was demonstrated in unpaired t-test (*p =* < 0.0001, CI 95% -0.55 to -0.40*) (*Fig. 7A*).*

### AlkaPhos compared to fluorescence in situ hybridization

For tumors with and without 1p deletion in fluorescence in situ hybridization, median fluorescence ratios ΔF570/480 of 0.31 and 0.53 were recorded, respectively. Normal distribution could not be assumed based on the results of Shapiro-Wilk test (1p deletion W = 0.95, *p* = 0.72; no 1p deletion W = 0.78, *p* = 0.04). Mann-Whitney-U test revealed no significant difference in median fluorescence ratios ΔF570/480 between tumors with and without 1p deletion (*p* = 0.22), but a clear trend towards higher fluorescence ratios in tumors without 1p deletion (Fig. 7B).

In 11/14 (78%) samples, matching results for 1p deletion or retention in FISH and negative or positive AlkaPhos results were obtained, respectively. AlkaPhos testing correctly identified 8/8 (100%) tumors with confirmed 1p deletion.

### AlkaPhos compared to loss of heterozygosity

For tumors with and without LOH of 1p, median fluorescence ratios ΔF570/480 of 0.29 and 0.33 were recorded, respectively. Normal distribution could not be assumed based on the results of Shapiro-Wilk test (LOH 1p W = 0.91, *p* = 0.46; no LOH 1p W = 0.77, *p* = 0.02). Mann-Whitney-U test revealed no significant difference in median fluorescence ratios ΔF570/480 between tumors with and without LOH of 1p (*p* = 0.20), but a trend towards higher fluorescence ratios in tumors without 1p deletion (Fig. 7C).

In 10/14 (71%) samples, matching results for LOH of 1p and negative or positive AlkaPhos results were obtained, respectively. AlkaPhos correctly identified 7/7 (100%) tumors with LOH of 1p.

### AlkaPhos compared to histochemical analysis

For tumors with and without presence of alkaline phosphatase in histochemical analysis, mean fluorescence ratios ΔF570/480 of 0.53 (SD 0.25) and 0.31 (SD 0.05) were recorded, respectively. Normal distribution was assumed based on the results of Shapiro-Wilk test (negative samples W = 0.86, *p* = 0.15; positive samples W = 0.81, *p* = 0.057). Unpaired t-test revealed a significant difference in mean fluorescence ratios ΔF570/480 between tumors with and without presence of alkaline phosphatase in histochemical analysis (*p* = 0.03, 95% CI -0.43 to -0.01) (Fig. 7D).

In 10/14 (71%) samples, matching results in histochemical analysis and AlkaPhos testing were obtained, respectively (Table 2). AlkaPhos never indicated presence of alkaline phosphatase without concomitant presence of alkaline phosphatase in histochemical analysis.

### Overall comparison of different methods

As presented in Table 2, AlkaPhos, FISH, LOH and histochemical analysis revealed matching results regarding 1p-status and alkaline phosphatase in only 8/14 (57%) of tumors.

While genetic results for 1p-status obtained with FISH and LOH were congruent in 13/14 tumors, a discrepancy was recorded in tumor T6. Here, FISH revealed 1p deletion, while no LOH of 1p was present. Both, AlkaPhos and histochemical testing for alkaline phosphatase, demonstrated absence of alkaline phosphatase in this tumor.

In tumor T12, genetic testing demonstrated 1p retention, while alkaline phosphatase could not be detected by AlkaPhos or histochemical testing.

In tumors T11 and T13, genetic testing demonstrated 1p retention, while alkaline phosphatase could not be detected by AlkaPhos, but histochemical testing indicated presence of alkaline phosphatase.

AlkaPhos testing and histochemical analysis both correlated to overall 1p-status in genetic testing in 11/14 (78%) cases. AlkaPhos correctly identified 8/8 (FISH) and 7/7 (LOH) (100%) tumors with 1p deletion and LOH of 1p, whereas histochemical analysis could only identify 6/8 (75%, FISH) and 5/7 (71%, LOH), respectively.

## Discussion

In this study, a proof-of-concept for a novel tool to report intracellular alkaline phosphatase presence in human meningioma cells is introduced. AlkaPhos could specifically indicate alkaline phosphatase activity in BEN-MEN-1 cell cultures. In primary meningioma cell cultures, AlkaPhos could sufficiently identify tumor cell cultures in which deletion of chromosome 1p in fluorescence in situ hybridization (FISH) or loss of heterozygosity (LOH) of 1p were present, respectively. AlkaPhos outperformed conventional histochemical analysis in correctly identifying tumors with 1p deletion and LOH of 1p by detecting alkaline phosphatase activity.

Deletion of the short arm of chromosome 1 (chromosome 1p), as an independent predictor for an increased risk of recurrence in meningioma patients, is present in about 9% of meningiomas [13, 18, 19, 21]. In this study, 8/14 (57%) tumors (FISH) and 7/14 (50%) tumors (LOH) with 1p deletion and LOH 1p were included in order to achieve a balanced distribution between tumors with and without these aberrations. Thus, the ratio of tumors with 1p deletion in this study was higher than in a representative cohort of consecutive patients [18]. 

The *alkaline phosphatase* gene is located in the chromosomal region 1p36.1-24 [14, 24]. A correlation of deletion of chromosome 1p and LOH of 1p with the complete absence of alkaline phosphatase activity in meningiomas is suspected to originate from gene silencing via submicroscopic alterations, mutations or genomic imprinting in the remaining gene [4, 23, 24]. 

To date, the gold standard to detect deletions of chromosome 1p is FISH given its superior sensitivity compared to conventional karyotyping [20]. Histochemical testing for alkaline phosphatase proved to serve as an additional indirect tool to detect deletions of chromosome 1p in meningiomas [4, 23, 24]. However, both methods are in need of the expertise, time and equipment of specialized laboratories and staff, such as fluorescently labeled DNA probes specific to the region of interest, sequencing or genotyping platforms for LOH detection, high-quality reagents for sample preparation, technicians trained in cytogenetics to conduct analyses and interpret results, excluding low-resource areas from performing these crucial diagnostics in meningiomas. The unique dual-emission characteristics of AlkaPhos, which allow substrate-product distinction, offer significant advantages over conventional methods. Its ratiometric fluorescence readout minimizes errors related to substrate concentration variability, making it suitable for broader applications, including low-resource settings where sophisticated infrastructure for FISH or LOH testing is unavailable [8]. 

In the current study, the ability of AlkaPhos to accurately distinguish between presence and absence of alkaline phosphatase in meningioma cells could be demonstrated on the human meningioma cell line BEN-MEN-1 before and after treatment with the alkaline phosphatase inhibitor L-p-bromotetramisole oxalate.

Applied on primary meningioma cell cultures, AlkaPhos demonstrated a robust read-out of alkaline phosphatase activity and sufficiently identified all tumors with confirmed 1p-deletion in FISH or LOH of 1p, but statistical significance for AlkaPhos to determine 1p-status was not reached. AlkaPhos was able to identify more cases in which 1p-deletion was confirmed with FISH or in which LOH of 1p was present, compared to histochemical detection of alkaline phosphatase.

Tumor T12 displayed no 1p deletion or LOH of 1p, but neither AlkaPhos nor conventional histochemical analysis demonstrated presence of alkaline phosphatase. Niedermayer et *al*. recognized an uncommon pericentric inversion of one chromosome 1 as the cause of alkaline phosphatase activity loss [24]. In the current study, no further genetic testing, e.g., exome sequencing or karyotyping, was performed on this tumor. However, a similar structural aberration as in the referenced case, untraceable by FISH or LOH, as well as epigenetic changes could also affect the alkaline phosphatase activity in the cases presented in our study.

Despite an intact chromosome 1p in FISH or LOH analysis, AlkaPhos did not show alkaline phosphatase activity in 3/6 (FISH) and 4/7 (LOH) cases, whereas conventional histological staining revealed enzyme activity. Intratumoral genetic heterogeneity of meningiomas as described by Urbschat et *al.* [33] could impact an uneven distribution of alkaline phosphatase activity throughout different areas of one tumor since different areas of one tumor were analyzed by the different testing modalities. Heterogeneity for loss of chromosome 1p or the above mentioned pericentric inversion could affect fluctuation of alkaline phosphatase activity in different areas of the tumor. Hence, inconsistent results for enzyme activity measured by AlkaPhos and conventional histological staining in the same tumor cannot be entirely excluded.

Besides 1p deletion, losses of chromosome 6q, 10, 17q and 18q, mutations of the *TERT* promoter or homozygous deletions of *CDKN2A/B* are described to influence the clinical course in meningioma patients [3, 5, 12, 30]. Genetic alterations in meningiomas have been implemented in the current WHO grading system, but require highly equipped and specialized oncologic centers to perform the appropriate testing [3, 29]. Simplified, yet robust testing options in a manner of point-of-care testing to detect genetic markers for an increased recurrence risk in meningiomas could ameliorate postoperative patient care in low-resource areas.

Point-of-care diagnostic tools have improved medical care especially in low-resource areas [11, 31]. Most point-of-care testing options are based on antibody, enzyme or receptor interactions, while genetic point-of-care or bedside testing has been routinely used in the CYP2C19*2 allele detection [11, 28] and molecular testing options such as microRNA testing are under development [6, 11, 28, 34]. AlkaPhos represents a novel potential agent for point-of-care testing in meningiomas based on enzyme activity detection. In this study, a proof-of-concept for the mechanism of AlkaPhos with reliable results in the detection of alkaline phosphatase activity, and hence 1p deletion in primary meningioma cell cultures, is presented.

Given that the AlkaPhos and FISH experiments were performed on primary cell cultures and not on native tumor tissue, the primary aspect of low-resource diagnostics is not yet accomplished. To further develop the application of AlkaPhos in routine clinical diagnostics in the future, a prospective study on freshly resected tumor samples is mandatory. The fluorescence emissions of both, the AlkaPhos substrate and product, are covered by the excitement range of near-ultraviolet (near-UV) light. Ideally, alkaline phosphatase activity could be detected intraoperatively with a fluorescence microscope, or even a smartphone-based system combined with near-UV light, on minced tumor samples [2, 27]. A comparison of AlkaPhos, histochemical alkaline phosphatase detection and fluorescence in situ hybridization analyses on native tumor tissue is planned to improve comparability between results and determine the qualities of AlkaPhos as a routine diagnostic tool.

Due to the small sample size of only 14 tumors, the statistical evaluation of the study at hand remains to be substantiated in a larger cohort. As of now, a statistical significance in differences of mean fluorescence values between tumors with and without 1p deletion or LOH of 1p is not yet reached, but a trend towards higher mean fluorescence values in tumors without 1p deletion or LOH of 1p was noted.

Perspectively, meningioma patients at high risk of tumor recurrence could be comprehensively and reliably identified at the earliest time point by AlkaPhos testing. For patients treated in low-resource areas, the possibility of fast, reliable and cost-effective genetic diagnostics to detect 1p deletion in meningiomas would ameliorate the postoperative consulting and follow-up regime and possibly lead to an early detection of tumor recurrence.

## Conclusion

With AlkaPhos, we present a fluorescent, ratiometric probe with the potential to become a point-of-care intraoperative diagnostic tool to test meningiomas for alkaline phosphatase activity and hence the loss of chromosome 1p in the tumor. The AlkaPhos emissions of substrate and product are distinguishable but insensitive to variations in substrate concentration and therefore superior to other fluorogenic substrates for measuring the enzymatic phosphatase activity in such heterogeneous environments as native tumor tissue. AlkaPhos could provide a diagnostic tool capable of quickly identifying meningioma patients at high risk for tumor recurrence independent from the WHO-grading of the tumor without complex genetic testing and thus guide the postoperative surveillance regime even in low-resource areas without access to advanced genetic testing.

The promising results of this study emphasize the importance for further deployment of AlkaPhos in clinical applications.

## Electronic supplementary material

Below is the link to the electronic supplementary material.

Supplementary Material 1

## Data Availability

Data is provided within the manuscript and the supplementary material file.
