# Supplementary material for: AlkaPhos: a novel fluorescent probe as a potential point-of-care diagnostic tool to estimate recurrence risk of meningiomas
Source: Neurosurg Rev. 2025 Jan 8;48(1):27. doi: 10.1007/s10143-024-03172-8 (PMC11706915; doi:10.1007/s10143-024-03172-8)
Supplement: Supplementary file 1 — Supplementary Material 1 [file 10143_2024_3172_MOESM1_ESM.pdf]

AlkaPhos: A Novel Fluorescent Probe as a Potential Point-of-Care Diagnostic Tool to Estimate Recurrence Risk of Meningiomas: Supplemental material

Journal: Neurosurgical Review

Sina Hemmer[1], Xin Hui[2,3], Julia Draeger[2], Johannes Menges[2], Eva C. Schwarz[4], Arne Wrede[5], Joachim Oertel[1], Lars Kaestner[3,6], Gregor Jung[2], Steffi Urbschat[1]

[1] Department of Neurosurgery, Saarland University Medical Center, Homburg, Germany (S.H., J.O., S.U.)

[2] Biophysical Chemistry, Saarland University, Saarbrücken, Germany (X.H., J.D., J.M., G.J.)

[3] Theoretical Medicine and Biosciences, Saarland University, Homburg, Germany (X.H., L.K.)

[4] Biophysics, Center for Integrative Physiology and Molecular Medicine, Saarland University, Homburg, Germany

[5] Institute for Neuropathology, Saarland University Medical Center, Homburg, Germany (A.W.)

[6] Experimental Physics, Saarland University, Saarbrücken, Germany (L.K.)

Correspondence to: Steffi Urbschat, Department of Neurosurgery, Building 90.5, Kirrbergerstraße 100, 66421 Homburg/Saar, phone: +49 6841 16 26635, fax: +49 6841 16 24480, e-mail: Steffi.urbschat@uks.eu

AlkaPhos

AlkaPhos (Bis(methylene)-diacetate-(3,6,8-tris(*N*-methoxy-*N*-methylsulfamoyl)pyren-1-yl)phosphate) is a synthesized cell permeable fluorescent probe to measure alkaline phosphatase activity.,[2, 3] Due to its ester groups, water soluble AlkaPhos **(1)** is membrane permeable, but once inside the cell the ester groups are cleaved. AlkaPhos **(1)** is intracellularly transformed into the actual phosphatase substrate (**2**) which accumulates due to its negative charges.[7] Alkaline phosphatase (from bovine intestinal mucosa) converts the phosphomonoester (**2**) in cuvette experiments approximately as fast as other widely used substrates (turn-over frequency k_{cat} (**2**) = $3*10^2$ s^{-1})[3–5] and its activity saturates at slightly higher substrate concentrations than the formerly introduced pyrene derivatives (Michaelis-Menten constant K_M (**2**) = $1*10^{-5}$ M at pH 8.2).[2] Comparison with the weaker binding and less active trisulfonated pyranine phosphate suggest that the higher K_M can be traced back to the better water solubility compared to previous compounds.[3, 6]

The substrate (**2**) is converted into (**3**) in the presence of alkaline phosphatase (*Figure 1*). Both substrates (**1**) and (**2**) and the product (**3**) show bright fluorescence (Φ_{Fl} = 35 ±5% (**1**), 80 ±5% (**2**), and 98% (**3**), respectively[3]) between pH 6 and 9 and can be easily distinguished by their electronic spectra ((**1**): λ_{abs} = 407 nm; λ_{em} = 473 nm; (**2**): λ_{abs} = 416 nm; λ_{em} = 477 nm; (**3**): λ_{abs} = 510 nm; λ_{em} = 557 nm at pH > 6).[3] Appropriate filter sets in fluorescence microscopy allow for recording the ratiometric response as result of enzymatic activity.

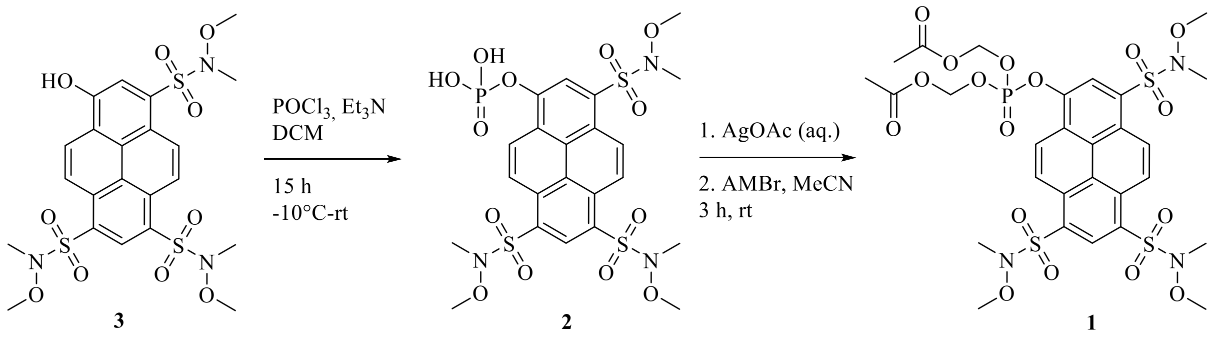

3 2 1

Fig. 1 *Synthesis of membrane permeable AlkaPhos (1) by phosphorylation (2) and esterification of the starting compound (3)* [1,2]

Chemicals and synthesis of AlkaPhos

Solvents and chemicals including alkaline phosphatase from bovine intestinal mucosa were obtained from Sigma-Aldrich except for dichloromethane (DCM; Fisher Scientific), and were used as received. Structural formulas of AlkaPhos **(1)**, its salt form **(2)** and the product **(3)** are given in *Figure 2*.

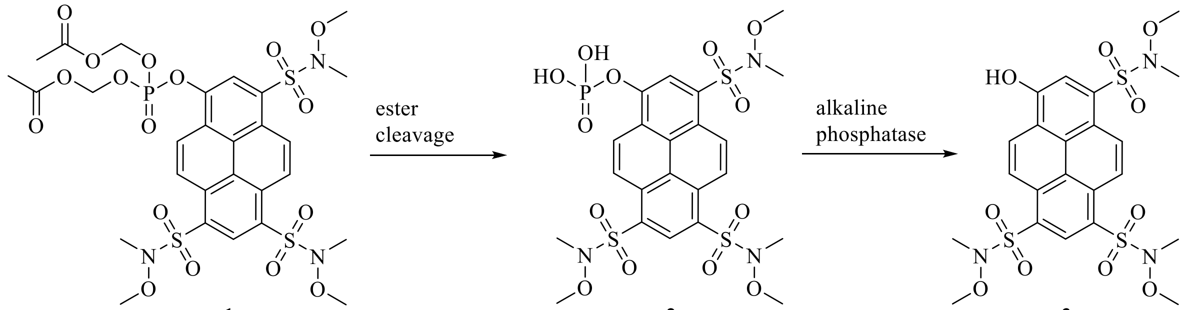

Fig. 2 *Conversion of AlkaPhos (**1**) by cleavage of the ester groups (**2**) and the phosphate group by alkaline phosphatase (3)*

Synthesis of ***(2)***

Phosphoroxy chloride ($POCl_3$; 271 µL, 2.90 mmol) was dissolved in DCM (40 mL) and cooled to -10 °C. A solution of 8-hydroxy-*N, N‘, N“*-trimethoxy-*N, N‘, N“*-trimethylpyrene-1,3,6-trisulfonamide[3] ((**3**); 96 mg, 163 µmol) and trimethylamine (Et_3N; 250 µL) in DCM (15 mL) was slowly added. The reaction mixture was stirred overnight at room temperature. Water was added and the organic layer was separated afterwards. The organic phase was washed twice with hydrochloric acid (1 M) and once with brine, dried over Na_2SO_4, then the solvent was removed in vacuo. The solid was dissolved in water (10 mL) for hydrolysis and stirred overnight at room temperature. The solution was concentrated in vacuo and the crude product (**2**) was used without further purification (80.9 mg, 121 µmol, 39 %).

^{1}H-NMR (500 MHz, D_2O): δ [ppm] = 2.69 (s, 3 H), 2.77 (s, 3 H), 2.85 (s, 3 H), 3.31 (s, 3 H), 3.36 (s, 3 H), 3.62 (s, 3 H), 8.59 (d, *J* = 10.0 Hz, 1 H), 8.67 (s, 1 H), 8.85 (s, 1 H), 8.89 (d, *J* = 9.8 Hz*,* 1 H), 8.93 (d, *J*= 9.8 Hz*,* 1 H), 8.94 (d, *J*= 9.8 Hz*,* 1 H).

^{13}C-NMR (125 MHz, MeOD-d$_4$): δ [ppm] = 38.5, 39.3, 64.0, 64.0, 64.4, 123.6, 123.8, 124.0, 124.1, 125.4, 125.8, 126.3, 127.0, 129.4, 129.8, 130.9, 134.5, 134.6, 135.9, 152.9.

^{31}P-NMR (203 MHz, D_2O): δ [ppm] = 0.93.

Synthesis of ***(1)***

3,6,8-tris-(N-methoxy-N-methylsulfamoyl)pyren-1-yl dihydrogen phosphate (65.0 mg, 97.4 µmol) (**2**) was dissolved in water, and an equal amount of a saturated solution of silver acetate with solid silver acetate was added. The precipitating silver salt of (**2**) (63.8 mg, 72.6 µmol, 75 %) was filtered off, washed with water and dried.

Subsequently, the silver phosphate was dissolved in dry acetonitrile (MeCN; 1 mL). Bromomethyl acetate (AMBr; 18.8 µL, 192 µmol) was added and the solution was stirred at room temperature for 3 h. After evaporation of the solvent, the solid was dissolved in dichloromethane and the residual, insoluble deposit was removed by filtration. NMR spectroscopy of the yellow product (**3**) (30.0 mg, 37 µmol, 51 %) revealed a purity of 97 %.

^{1}H-NMR (400 MHz, $CDCl_3$): δ = 2.09 (s, 6 H), 2.91-2.93 (m, 9 H), 3.78-3.83 (m, 9 H), 5.77-5.86 (m, 4 H), 8.82 (d, 1 H), 8.83 (s, 1 H), 9.37 (s, 1 H), 9.48 (d, 1 H), 9.57 (d,1 H), 9.61 (d, 1 H).

^{13}C-NMR (100 MHz, $CDCl_3$): δ = 20.4,38.7, 38.7, 38.9, 63.5, 63.5, 63.7, 83.1, 83.2, 122.8, 122.9, 125.1, 125.3, 125.3, 126.0, 126.1, 126.6, 126.7, 127.5, 128.3, 129.4, 129.4, 129.6, 133.9, 134.3, 134.6, 144.6, 169.0.

^{31}P-NMR (162 MHz, $CDCl_3$): δ [ppm] = -9.02.

AlkaPhos spectra determination and calibration

Spectra determination

First, optical spectra and the *in vitro* kinetics were recorded by absorption (Jasco, V-650) or fluorescence spectroscopy (Jasco, FP-6500). Fluorescence quantum yields determinations were performed in a quantum yield spectrometer (Hamamatsu, C11347). ^{1}H-, ^{13}C and ^{31}P-NMR spectra were recorded with a Bruker AM 400 or Bruker AM 500 spectrometer.

Quantum yields determinations revealed the fluorescence spectra of AlkaPhos at the two spectral channels F480 and F570 (*Figure 1A of the main manuscript*).

Determination of in vitro dephosphorylation

Second, determination of the *in vitro* dephosphorylation of AlkaPhos was conducted. The salt form of AlkaPhos, (**2**) was dissolved in Tyrode's solution (135 mM NaCl, 5.4 mM KCl, 1.8 mM $CaCl_2$, 10 mM glucose, 2 mM $MgCl_2$ and 10 mM HEPES; pH 7.35) at desired working concentration. The measurements were performed on an inverted microscope (ECLIPSE Ti, Nikon, Japan) with a 20x objective (NA 0.75, Nikon, Japan) as previously described.[1] The microscope was attached to a Nipkow disk scanner (CSU-W1, Yokogawa, Japan). Two solid-state lasers at 442 nm and 515 nm wavelength worked as light sources. The emission fluorescence was separated through a dichroic mirror (491 nm) and recorded on two spectrally separated channels (480 ± 40 nm and 570 ± 40 nm) by an sCMOS camera (Flash4, Hamamatsu, Japan). In vitro measurements were performed by application of 0.4 µg/mL alkaline phosphatase (Merck, Germany) into the AlkaPhos solution at pointed time and the fluorescent images (512 × 512 pixels) were recorded at 0.5 frames per second. The acquired images were analyzed by ImageJ (National Institute of Health, USA) to correct the image background and collect the fluorescence intensity in regions of interest over time.

References

1. Abay A, Simionato G, Chachanidze R, Bogdanova A, Hertz L, Bianchi P, Van Den Akker E, Von Lindern M, Leonetti M, Minetti G, Wagner C, Kaestner L (2019) Glutaraldehyde - A subtle tool in the investigation of healthy and pathologic red blood cells. Front Physiol 10:1–14. doi: 10.3389/fphys.2019.00514
2. Finkler B, Riemann I, Vester M, Grüter A, Stracke F, Jung G (2016) Monomolecular pyrenol-derivatives as multi-emissive probes for orthogonal reactivities. Photochem Photobiol Sci 15:1544–1557. doi: 10.1039/C6PP00290K
3. Finkler B, Spies C, Vester M, Walte F, Omlor K, Riemann I, Zimmer M, Stracke F, Gerhards M, Jung G (2014) Highly photostable "super"-photoacids for ultrasensitive fluorescence spectroscopy. Photochemical and Photobiological Sciences 13:548–562. doi: 10.1039/c3pp50404b
4. Levine MN, Raines RT (2011) Sensitive fluorogenic substrate for alkaline phosphatase. Anal Biochem 418:247–252. doi: 10.1016/j.ab.2011.07.021
5. Obayashi Y, Iino R, Noji H (2015) A single-molecule digital enzyme assay using alkaline phosphatase with a cumarin-based fluorogenic substrate. Analyst 140:5065–5073. doi: 10.1039/c5an00714c
6. Sato E, Chiba K, Hoshi M, Kanaoka Y (1992) Pyranine Phosphate as a New Fluorogenic Substrate for Acidic and Alkaline Phosphatase. Chem Pharm Bull (Tokyo) 40:786–788
7. Schultz C, Vajanaphanich M, Harootunian AT, Sammak PJ, Barrett KE, Tsien RY (1993) Acetoxymethyl esters of phosphates, enhancement of the permeability and potency of cAMP. Journal of Biological Chemistry 268:6316–6322. doi: 10.1016/S0021-9258(18)53255-5
